# Enteric pathogen infection and consequences for child growth in young Aboriginal Australian children: a cross-sectional study

**DOI:** 10.1186/s12879-020-05685-1

**Published:** 2021-01-06

**Authors:** Sarah Hanieh, Siddhartha Mahanty, George Gurruwiwi, Therese Kearns, Roslyn Dhurrkay, Veronica Gondarra, Jenny Shield, Norbert Ryan, Francesca Azzato, Susan A. Ballard, Nicole Orlando, Sullen Nicholson, Katherine Gibney, Julie Brimblecombe, Wendy Page, Leonard C. Harrison, Beverley-Ann Biggs, on behalf of the Child Health and Nutrition Study team, Yalurr Dhamarandji, David Djilimara, Elizabeth Bungawara, Lloyd Dhamarandji, Janice Djiliri, Jess Gatti, Jannie Kraayenhof, Noella Goveas

**Affiliations:** 1 Department of Medicine at the Peter Doherty Institute for Infection and Immunity, University of Melbourne, Melbourne, Victoria Australia; 2 The Victorian Infectious Diseases Service, Royal Melbourne Hospital, Parkville, Victoria 3052 Australia; 3 Menzies School of Health Research, Darwin, Northern Territory 0810 Australia; 4 Victorian Infectious Diseases Reference Laboratory, Peter Doherty Institute, Melbourne, Victoria Australia; 5 Microbiological Diagnostic Unit Public Health Laboratory at the University of Melbourne, The Peter Doherty Institute for Infection and Immunity, Melbourne, Australia; 6 Department of Nutrition, Dietetics and Food, Monash University, Clayton, Victoria Australia; 7 Miwatj Health Aboriginal Corporation, Nhulunbuy, NT 0881 Australia; 8 Public Health and Tropical Medicine, James Cook University, Cairns, QLD 4870 Australia; 9 Walter and Eliza Hall Institute of Medical Research, Parkville, VIC Australia

**Keywords:** Enteric infection, Child growth, Aboriginal, Height for age z scores

## Abstract

**Background:**

To determine the prevalence of enteric infections in Aboriginal children aged 0–2 years using conventional and molecular diagnostic techniques and to explore associations between the presence of pathogens and child growth.

**Methods:**

Cross-sectional analysis of Aboriginal children (*n* = 62) residing in a remote community in Northern Australia, conducted from July 24th - October 30th 2017. Stool samples were analysed for organisms by microscopy (directly in the field and following fixation and storage in sodium-acetate formalin), and by qualitative PCR for viruses, bacteria and parasites and serology for *Strongyloides*-specific IgG. Child growth (height and weight) was measured and z scores calculated according to WHO growth standards.

**Results:**

Nearly 60% of children had evidence for at least one enteric pathogen in their stool (37/62). The highest burden of infection was with adenovirus/sapovirus (22.9%), followed by astrovirus (9.8%) and *Cryptosporidium hominis/parvum* (8.2%). Non-pathogenic organisms were detected in 22.5% of children. Ten percent of children had diarrhea at the time of stool collection. Infection with two or more pathogens was negatively associated with height for age z scores (− 1.34, 95% CI − 2.61 to − 0.07), as was carriage of the non-pathogen *Blastocystis hominis* (− 2.05, 95% CI - 3.55 to − 0.54).

**Conclusions:**

Infants and toddlers living in this remote Northern Australian Aboriginal community had a high burden of enteric pathogens and non-pathogens. The association between carriage of pathogens/non-pathogens with impaired child growth in the critical first 1000 days of life has implications for healthy child growth and development and warrants further investigation. These findings have relevance for many other First Nations Communities that face many of the same challenges with regard to poverty, infections, and malnutrition.

**Supplementary Information:**

The online version contains supplementary material available at 10.1186/s12879-020-05685-1.

## Background

Infections with enteric pathogens are common in disadvantaged populations with low socio-economic status due to inadequate sanitation and hygiene, overcrowded living conditions, and gaps in health literacy [1]. During the first two years of life, enteric infections have a particularly devastating effect on child health as a result of poor nutritional status, loss of appetite and diarrhea, disturbances in intestinal function that lead to impaired absorption of micronutrients and low grade inflammation [2]. More specifically, infection with *Shigella sp.and Giardia lamblia* have been associated with a significantly higher risk of stunting [2]; enterotoxigenic *Escherichia coli* with a higher risk of being underweight [3] *Campylobacter jejuni* has been shown to be associated with lower growth rates for both length and weight [4]; and hookworm and *Trichuris trichiura* have been associated with anaemia [5, 6]. As well as impaired growth and cognitive development, children may have anaemia and experience lethargy, which can result in reduced educational achievement and decreased opportunities in later life [7, 8]. Impaired child growth in the first 1000 days of life is also associated with long-lasting adverse consequences for adult health, including increased risk of chronic diseases [8–10].

A high prevalence of enteric infections has been previously reported in Australian Aboriginal communities, from a small number of surveys in older children and adults [11–15]. However there is a paucity of studies of enteric infections in very young children in these communities. This is mainly due to difficulties in collecting and transporting samples from isolated locations, the limitations of conventional methodology, [11, 12] and challenges in achieving an accurate diagnosis in a field setting. New technologies such as multiplex molecular assays now allow more rapid and sensitive and simultaneous diagnosis of multiple enteric organisms [16] and more reliable diagnosis in remote settings. Recent studies suggest that “silent” enteric infections, in the absence of overt diarrhea, may still result in significantly adverse clinical outcomes, such as growth faltering [17–19]. The goal of our study was to determine: i) the prevalence of enteric carriage in all Aboriginal children aged between 0 and 2 years living in a remote Northern Australian community using conventional and molecular techniques, and ii) the association between the presence of enteric pathogens/non-pathogens and child growth outcomes.

## Methods

### Study design

We conducted a cross-sectional study of children residing in a remote Aboriginal community in Northern Australia who were between 0 and 2 years of age. Primary outcomes were i) prevalence of enteric infections (bacterial, viral, parasitic) using microscopy, PCR and serology; ii) child growth outcomes (height for age, weight for age, and weight for height z scores).

### Study setting and participants

The study was conducted in a remote tropical Northern Territory community between July 24th and October 30th 2017. All Aboriginal children aged between 0 and 2 years who were resident in the local community during this period (n ~ 100) and had primary carers who gave consent for participation in the study were eligible for enrolment in the study. Information relating to socio-demographic and food security data were collected via questionnaire, and anthropometric measurements were performed. Fresh stool samples were collected from the child’s nappy.

### Data collection and analysis

The field team consisted of two Aboriginal Health Practitioners, five Aboriginal Community Based Researchers, two parasitologists and three research scientists (including a paediatrician). According to information from the health services, it was estimated that 100 eligible children aged between 0 and 2 years of age were currently resident in the community.

Prior to commencement of the study, the local research team received a one week training course which included information on the study protocol; recruitment of participants and consent; telling of the research story in the local languages; data collection techniques (including the use of mobile devices for data collection and use of Research Electronic Data Capture - RedCap); faecal parasites and microscope literacy; anthropometric measurements; and assistance with blood collection (venous and Haemacue® machine). Eligible children were identified through a number of sources including the local health clinics, day care centre, local play group, community child nutrition programs, local Aboriginal researcher contacts and by visiting individual houses.

#### Maternal characteristics

Trained research staff collected maternal socio-demographic, nutritional, environmental, wealth index, breast feeding patterns and food security information via RedCap using a structured questionnaire (Supplementary 1). The questionnaire was initially tested in a focus group session among community members and local staff during the training course, and refined according to local community feedback. An information sheet including pictorial information was prepared by the research team, refined according to community feedback and then translated into local language by Aboriginal researchers.

#### Anthropometric measurements

Child anthropometric measurements (height/length and weight) were measured on enrolment using a portable infantometer (Shorr Board®) for length and standard anthropometer for height. A digital infant weighing scale/ or mother-infant scale (Secca 876) with precision to the nearest 10 g was used to measure weight. A mother-Infant scale (Secca 876) was used to determine maternal weights. Height was measured to the nearest 0.5 cm using a standard anthropometer. Maternal body mass index was calculated using maternal height and weight (kg/m^2^) Child weight for age, weight for length and length for age z scores were calculated using WHO Anthro (version 3.2.2, January 2011) [20]. Stunting was defined as height or length-for-age z scores less than two standard deviations below WHO Child Growth Standards [20].

#### Wealth Index

To construct a wealth index for the infant’s household, principal component analysis was used from the items belonging to the following three component indices: i) housing quality (three items: average number of persons per room [number], floor [five responses], wall [four responses]), ii) consumer durables (four items [yes/no]: fridge, television, car, phone), and iii) cooking energy [three responses]. The wealth index score was grouped into tertiles from tertile 1 (poorest) to tertile 3 (richest) [21].

#### Clinical examination

Following enrolment into the study each child received a general health check by a paediatrician. This included clinical history of diarrhea at presentation (defined as maternal report of three or more loose stools in 24 h), and clinical examination for signs of infection (respiratory, skin, ears) [22]. If signs of infection were detected on clinical examination, the child was referred to the local clinic for further management. Children with infection were included in the analysis.

#### Stool collection and analysis

Primary carers were instructed to deliver soiled nappies to the study field laboratory or telephone the research team who collected them within two hours. Fresh stool was examined as soon as possible by direct microscopy and Kato-Katz, and participants with enteric infections were referred to the health clinic for further management. Of the remaining sample, an aliquot was stored in 10 ml of sodium acetate formalin (SAF) solution (Bioline), and another frozen at − 20 °C for PCR analysis. Stool samples collected into SAF were sent to the Victorian Infectious Diseases Reference Laboratory, The Peter Doherty Institute for Infection and Immunity, Melbourne, Australia for testing using the formalin ethyl acetate (FEA) concentration method [23]. Stained fixed smear and modified acid-fast staining methods were also applied.

### PCR detection of stool pathogens and non-pathogens

DNA was extracted from stool samples using the MolBio Powersoil® kit (QIAgen, Melbourne, Australia) for faecal pathogens and soil-transmitted helminths. DNA concentration was measured using Quant-iT™ dsDNA HS assay kit (Life Technologies, Australia) and normalized to 1 ng/μl in UltraPure DNase/RNase-free distilled water (Invitrogen). DNA samples (10 μl) were tested using the Tandem- Plex™ Fecal Pathogens M kit (Ausdiagnostics, Sydney, Australia) as described by the manufacturer Bacteria and viruses tested for are listed in Supplementary 2. An aliquot of the DNA extracted using the MolBio Powersoil Kit® was tested using the Tandem Plex™ Soil Transmitted Helminths kit (Ausdiagnostics) for the following soil-transmitted helminths: *Ascaris lumbricoides*, *Necator americanus*, *Ancylostoma ceylanicum*, *Ancylostoma duodenale* (“pan-hookworm”), *Trichuris trichiura* and *Strongyloides stercoralis*. In addition, DNA was extracted from 0.2 g of fresh stool sample on the Freedom Evo 100® (Tecan. Swithzerland) robotics system using the NucleoMag^R^ Virus Kit (Macherey Nagel, Germany) as per manufacturer’s instructions. Prior to DNA extraction on the Freedom Evo 100®, a processing step of mechanical lysis as described by Mu, et al [24] was performed with the following modification: Lysis Matrix E (MP Biomedicals) 2 ml tubes were used. DNA extracts were also tested in the Allplex™ GI-Helminth(I) Assay® (Seegene Inc., Seoul, Korea) as per manufacturer’s instructions. The panel of targets tested was as follows: *Hymenolepis species* (re-classified as *Rodentolepis species*), *Enterocytozoon/Encephalitozoon species*, *Taenia species*, *Strongyloides species*, *Ascaris species*, *Trichuris trichiura*, *Ancylostoma species* and *Necator americanus.* The limits of detection of the assay for the selected targets is reported to range between 17 and 93 copies/10uL by the manufacturer, [25] but is likely higher in our study due to modifications to the recommended DNA extraction procedures described above. Non-pathogens are defined as enteric parasites which do not cause disease. Included in this list were *Blastocystis hominis, Entamoeba coli, Iodamoeba butschlii, Chilomastix mesnili.*

### Serodiagnosis of strongyloidiasis

*Strongyloides* infections were diagnosed serologically using a commercially available ELISA that detects IgG antibodies against NIE, a recombinant protein from *S. stercoralis,* following kit manufacturer’s instructions (InBios, Inc., Seattle, USA). Positive and negative controls were tested in duplicate and patient serum samples tested singly. Control sera and sera from each subject were diluted to 1/100 using sample diluent buffer. It should be noted the kit manufacturer does not provide a cut- off value. As stated by the manufacturer the ‘cut-off value will vary depending on the test population and geographical location where the kit is being used.’ We calculated a cut-off value using ROC analysis of 98 sera collected in Australia. The results are reported qualitatively [26].

### Statistical analysis

Data was analyzed using StataIC, version 14 (StataCorp, College Station, TX, USA). Categorical data are presented as percentages with frequency, and continuous data are presented as mean and standard deviation (SD). Multivariate linear (height for age z scores) and logistic regression (stunting) was performed to examine the association between prevalence of enteric infection and child growth outcomes. Unadjusted and adjusted estimates of mean differences or odds ratios, and their 95% confidence intervals (CI) are presented. Potential confounding factors were selected a priori. All variables were included and adjusted for in the final model.

## Results

We enrolled 70 of the ~ 100 children aged between 0 and 2 years resident in the community. Stool samples were collected from 62 (88.5%) children. In the sample of children who had stool samples collected, more than half of mothers/carers (51.6%) were educated to year 11 level. The prevalence of maternal underweight was 23%, overweight 19.4%, and obesity 8.1%. A smoker resided in 87.1% of households, and the average number of residents per household was six. Traditional medicine was used by 70.9% of families. The majority of children (87.1%) were currently breastfed. Food insecurity was common with 58.1% of households reporting running out of food in the previous two weeks. Almost all women (98.4%) received government benefits. Baseline characteristics are shown in Table 1.

**Table 1:** Baseline characteristic of mothers and children in a remote Aboriginal community (*n* = 62)

Maternal/primary carer characteristics	Number (%) or Mean [SD] or Median {25th to 75th centile}
Grade of education	
Year 8–10	15/ 62(24.2)
Year 11	32/62 (51.6)
Year 12	15/ 62 (24.2)
Job^a^	
Receiving unemployment benefits	61/62 (98.4)
Employed	5/62 (8.6)
Body mass index	
Underweight (BMI < 18.5)	14/62 (22.6)
Normal (18.5 to 24.9)	31/62 (50)
Overweight (> = 25)	12/62 (19.4)
Obese (> = 30)	5/62 (8.1)
Mid-upper arm circumference (cm)^b^	23.7[3.96]
**Environmental**	
Presence of dogs in the house	34/62 (54.8)
Smoker in the house	54/62 (87.1)
Household ran out of food in the last 2 weeks	36/62 (58.1)
Child missed a meal in last 2 weeks	29/62 (46.8)
Presence of fridge in house	54/62 (87.1)
Number of people living in the house	6.3 [7.4]
Number of bedrooms in the house	3.3 [1.4]
Attendance at playgroup	49/62 (79.0)
Use of traditional medicine	44/62 (70.9)
Number of times mother washes hands/day	
1–2 times/day	45 /62(72.6)
> 2 times/day	17/62 (27.4)
Child uses soap	48/62 (77.4)
**Child characteristics**	
Sex	
Male	31/62 (50)
Female	31/62 (50)
Age (months)	10.6 {6.4 to 15.3}
Child currently breast fed	54/62 (87.1)
Exclusively breast fed	40/62 (64.5.)
Child ever bottle fed	22/62 (35.5)
Age complementary feeding started (months)	5.5 [1.3]

^a^Answer may be both

^b^ Six cases have missing data

### Prevalence of enteric infections

The prevalence of enteric infections is presented in Table 2. Overall prevalence of pathogen carriage was 59.7% (37/62) (i.e 37 out of 62 children had at least one pathogen detected), and three children were infected with three or more pathogens. More parasites were detected by microscopy of SAF samples (15 isolates from 62 samples) than direct stool microcopy (6/62). . Twenty- five children had no pathogens detected by any method.

**Table 2:** Prevalence of pathogenic versus non-pathogenic enteric carriage in young Aboriginal children residing in a remote community

PATHOGEN	Microscopy (***n*** = 62)		PCR (***n*** = 61)	
	Direct smear	SAF^a^	Ausdiagnostics	Allplex
**Bacteria**				
*Salmonella*	N/A	N/A	2 (3.3)	N/A
*Clostridium difficile*	N/A	N/A	1 (1.6)	N/A
*Campylobacter*	N/A	N/A	1 (1.6)	N/A
*Shigella*	N/A	N/A	4 (6.6)	N/A
**TOTAL**	**N/A**	**N/A**	**8 (13.1)**	**N/A**
**Virus**				
Adenovirus /Sapovirus	N/A	N/A	14 (22.9)	N/A
Astrovirus	N/A	N/A	6 (9.8)	N/A
Norovirus	N/A	N/A	3 (4.9)	N/A
Rotavirus	N/A	N/A	3 (4.9)	N/A
**TOTAL**	**N/A**	**N/A**	**24 (39.3)**	**N/A**
**Parasite**				
**Protozoa**				
*Giardia*	3 (4.8)	3 (4.8)	4 (6.6)	0 (0)
*Cryptosporidium*	0(0)	1 (1.6)	5 (8.2)	0 (0)
*Dientamoeba fragilis*	0(0)	4 (6.5)	N/A	0 (0)
**Helminths**				
*Trichuris trichiura*	2 (3.3)	4 (6.5)	N/A	3 (4.9)
*Strongyloides stercoralis*	0(0)	0(0)	N/A	0 (0)
*Enterobius vermicularis*	0(0)	1 (1.6)	N/A	5 (8.2)
***Cestodes***				
*Rodentolepis nana*	1 (1.6)	2(3.2)	N/A	2 (3.3)
**TOTAL**	**6 (9.7)**	**15 (24.6)**	**9 (14.8)**	**10 (16.4)**
**NON- PATHOGENS**				
**Parasite**				
*Blastocystis hominis*	7 (11.3)	8 (12.9)	N/A	N/A
*Entamoeba coli*	2 (3.2)	3(4.8)	N/A	N/A
*Iodamoeba butschlii*	0(0)	1 (1.6)	N/A	N/A
*Chilomastix mesnili*	1 (1.6)	1(1.6)	N/A	N/A
*Endolimax nana*	0(0)	1(1.6)	N/A	N/A
**TOTAL**	**10 (16.1)**	**12 (19.4)**	**N/A**	**N/A**

^a^Using techniques of either FE Concentrate, fixed smear or modified AF stain

Of the 24 children who had an enteric viral infection, eleven (47.8%) were co -infected with another pathogen, including *Cryptosporidium, Shigella*, *Salmonella*, *Giardia and Enterobius vermicularis*. The most commonly detected pathogen was adenovirus/sapovirus (14/61 samples tested; the two virus species are not distinguishable by the primers used in this kit), followed by astrovirus (6/61) *and Cryptosporidium hominis/parvum (*5/61). No cases of strongyloidiasis infection were detected by stool testing or serology (53 serology samples tested). No cases of hookworm were detected. Non-pathogens were detected in 14/62 (22.5%) of children, and in 10 cases coexisted with a pathogen. Ninety two percent of pathogen carriage was asymptomatic, with no reports of diarrhea or fever on clinical history or examination. Of the seven children who presented with diarrhea at the time of stool collection, pathogens were identified in four stool samples and included adenovirus, *Giardia lamblia, Rodentolepsis nana (R.nana), Trichuris trichiura*, and *Dientamoeba fragilis* and *Blastocystis hominis*.

### Association between enteric infection and child growth outcomes

Univariate and multivariate regression analyses to determine associations between pathogen and non-pathogen carriage and growth outcomes are presented in Table 3. Infection with two or more pathogens was found to negatively influence height for age z scores in young children (− 1.34, 95% CI − 2.61 to − 0.07), but not weight for age or weight for height z scores. No association was seen with risk of stunting. Infection separately with a bacterial, viral or parasitic infection was not associated with child growth outcomes. Infection with *Blastocystis* alone was also associated with reduced height for age z scores (− 2.05, 95% CI − 3.55 to − 0.54).

**Table 3:** Association between enteric carriage and child growth outcomes in a remote Indigenous community

	**Height for age z score**			
	**Univariable**		**Multivariable**^a^	
**Pathogen infection**	**Coeff (95% CI)**	***P*** **value**	**Coeff (95% CI)**	***P*** **value**
Bacterial infection	− 1.26 (−2.90 to 0.39)	0.13	− 1.61 (−3.36 to 0.12)	0.07
Viral infection	0.07 (−1.09 to 1.25)	0.89	0.02(−1.28 to 1.32)	0.97
Parasite infection	−1.022 (−2.23 to 0.24)	0.11	−1.21 (−2.51 to 0.08)	0.07
Infection with one pathogen	0.56 (−0.62 to 1.74)	0.35	0.59 (−0.64 to 1.82)	0.34
Infection with two or more pathogens	−0.97 (− 2.10 to 0.26)	0.12	−1.34 (−2.61 to − 0.07)	**0.04**
Non-pathogen demonstrated in stool	− 1.67 (− 2.93 to − 0.41)	**0.01**	− 1.83 (− 3.15 to − 0.50)	**0.01**
Blastocystis hominis detected in stool	−1.79 (− 3.24 to − 0.35)	**0.02**	−2.05 (− 3.55 to − 0.54)	**0.01**
	**Stunted**			
	**Univariable**		**Multivariable**	
	**OR (95% CI)**	***P*** **value**	**OR (95% CI)**	***P*** **value**
Bacterial infection	1.63 (0.28 to 9.41)	0.56	2.48 (0.32 to 19.36)	0.39
Viral infection	3.72 (0.95 to 14.56)	0.06	2.78 (0.60 to 12.92)	0.19
Parasite infection	1.07 (0.25 to 4.64)	0.93	1.02 (0.21 to 5.24)	0.98
Infection with one pathogen	3.17 (0.83 to 12.07)	0.09	3.16 (0.72 to 13.76)	0.13
Infection with two or more pathogens	1.36 (0.31 to 6.02)	0.68	1.11 (0.22 to 5.63)	0.90
Non-pathogen demonstrated in stool	2.34 (0.57 to 9.56)	0.23	3.92 (0.71 to 21.62)	0.12

^a^ Adjusted for wealth index, presence of dogs in house, handwashing frequency and maternal body mass index, and number of rooms in house

## Discussion

To our knowledge, this is the largest survey of young children aged 0–2 years in a remote Aboriginal community to document the prevalence of enteric infections and impact on child growth outcomes, and the first to compare different methodologies for identification of pathogens in this remote setting. We found that almost 60% of young children were infected with at least one enteric pathogen, most commonly adenovirus/sapovirus and *Cryptosporidium*, and infections with two or more pathogens was associated with impaired linear growth.

There is a paucity of data on the prevalence of enteric pathogen infections in infants and toddlers living in remote Aboriginal communities. We found only two studies in a remote Aboriginal community in the Northern Territory which documented the presence of intestinal parasites in 0–2 year old children [12]. One study in 1994–1996 at the same location identified the presence of *Ancylostoma. duodenale (A.duodenale)* (9%), *T.trichiura* (36%), *R.nana* (9%) and *Entamoeba spp (*9%). in < 24 months old children (*n* = 11), and *S. stercoralis* (27%), *R. nana* (17%), *Giardia duodenalis* (13%), *Entamoeba* spp. (10%), *A. duodenale* (13%), and *T.trichiura* (87%) in children aged 2–4 years 11 months (*n* = 30) between July 1994 to October 1996 [12]. Another study in the same location between 2010 and 2011 in children aged < 24 months demonstrated a prevalence of *S.stercoralis (*3% on microscopy (*n* = 29)*,* 9% on agar plate culture (*n* = 23), 8% on PCR [11] (personal communication Dr. Deborah Holt Sept 2019), 28% on serology (*n* = 38) [27] (personal communication Dr. Kate Mounsey Sept 2019), *T.trichiura* (14% on microscopy) and *R.nana* (7% on microscopy) (*n* = 29) [11] These findings are in contrast to those of our study, in which no cases of strongyloidiasis or hookworm were observed and the percentage of *T. trichiura* and *R. nana* positive were reduced. This reflects the implementation of public health measures (preventive chemotherapy, test and treat strategies) in this area that were introduced for soil transmitted helminths (albendazole from 1995, and an ivermectin mass drug administration in 2010 and 2011), leading to a reduction in *S. stercoralis* and hookworm prevalence [11, 15]. Another study examined long-term cryptosporidiosis patterns across Western Australia between 2002 and 2012, using data obtained from the Western Australian Notifiable Infectious Disease Database, and demonstrated a high burden of cryptosporidiosis in Aboriginal children aged 0–4 years of age (33.3%) [28]. Specific practices and environmental factors (e.g. hygiene and socioeconomic factors, presence of livestock and other animals, previous de-worming programs, weather patterns) may account for the different rate in our study, in which there were only five (8.2%) isolates of cryptosporidium.

There is growing evidence of the importance of pathogens in a child’s gut, even when asymptomatic. This “pathobiome” is thought to be causal in the development of ‘environmental enteropathy’, with associated chronic intestinal inflammation, villous blunting and intestinal leakage and malabsorption, leading to a cycle of malnutrition, micronutrient deficiencies, and growth impairment [29]. Childhood diarrhoea has been conceptualized as a syndrome of enteropathogen excess, rather than a single infection, with diarrhoea more likely to occur when a quantitative threshold of pathogen burden is exceeded [30]. Indeed in our study, the majority of children did not present with diarrhoea, and pathogen burden was likely to be low (as most isolates were only identified by PCR and diarrhoea was uncommon); however carriage of two or more enteric pathogens or *Blastocystis hominis* was associated with impaired linear growth (lower height for age z scores). This confirms that pathogens not causing diarrhoea may still cause significant morbidity in resource- constrained settings, and efforts towards improving environmental conditions including improvements in clean drinking water, housing standards and hygiene and sanitation are critical.

*B.hominis* is usually considered a non-pathogen but in some instances has been reported as a pathogen [31]. We observed a significant association between carriage of *B. hominis* and lower height for age z scores. This has been documented previously, with infection with *B. hominis* shown to be associated with lower anthropometric indexes in young children in Turkey [32]. These findings suggest that organisms characterised as ‘non-pathogenic’ may in fact carry some pathogenic potential, possibly through disturbance of the gut microbiome and/or damage to the intestinal mucosa resulting in increased intestinal permeability and impaired nutrient absorption [28]. Poor nutritional status may also have preceded infection by *B.hominis* [33].. The finding of *B. hominis* in stool may also represent exposure to organisms spread by the faecal-oral route [34].

Our study also provides important evidence on the application of molecular diagnostics to identify enteric pathogen burden. Use of PCR has previously been shown to substantially alter previous estimates of pathogen prevalence. Re-analysis of specimens from the MAL-ED cohort study using quantitative PCR demonstrated a predominance of viral causes of diarrhea (including sapovirus, rotavirus and adenovirus) with only two pathogens (rotavirus and enterotoxigenic *E. coli*) remaining in the top five causes of diarrhea identified by the original microbiological work up [35]. In our study, the majority of pathogens in stool samples from children were detected by PCR (qualitative), allowing increased detection rates and correlation with clinical outcomes.

Strengths of this study are that we worked closely with the local primary health care providers and trained Aboriginal Community Based Researchers to carry out the research, resulting in a high representative sample of young children in this remote Aboriginal community. We were also able to collect comprehensive information on possible nutritional, environmental and socio-demographic influences on clinical outcomes. Limitations are the relatively small sample size (even though it represented 68% of the target population), stool samples were not collected from mothers or carers of the child and hygiene practices being assessed only by questionnaire and not by direct observation (which may have introduced some recall bias); and we only asked about diarrheal infections in the last 24 h.

## Conclusions

We found a high prevalence of enteric pathogen and non-pathogen carriage in Australian Aboriginal infants and toddlers and a significant association between infection with multiple pathogens and child growth (impaired height for age z scores). These findings suggest that environmental contamination in remote Northern communities where extreme heat and humidity is common, may contribute to the high rates of childhood growth impairment and stunting reported, [36] with health implications extending into adulthood. The findings highlight the urgent need for sustainable strategies to improve housing, hygiene and sanitation in remote Aboriginal communities. Our findings also have relevance for many other First Nations Communities around the world that face similar challenges with regard to poverty, infections, and malnutrition.

## Supplementary Information

**Additional file 1.**

**Additional file 2.**

## Data Availability

Study data and materials may be made available from the corresponding author on request with appropriate human research ethics committee approval and with the consent of the participating community as required by Australian criteria for research with Indigenous communities.

## Abbreviations

*A. duodenale*: *Ancylostoma duodenale*
*B.hominis*: *Blastocystis hominis*
BMI: Body Mass Index
cm: centimetre
FEA: formalin ethyl acetate
kg: kilogram
*R. nana*: *Rodentolepis nana*
SAF: sodium acetate formalin
SD: standard deviation
*S.stercoralis*: *Strongyloides stercoralis*
*T.trichiura*: *Trichuris trichiura*
WHO: World Health Organization
