# Supplementary material for: Enteric pathogen infection and consequences for child growth in young Aboriginal Australian children: a cross-sectional study
Source: BMC Infect Dis. 2021 Jan 6;21:9. doi: 10.1186/s12879-020-05685-1 (PMC7788727; doi:10.1186/s12879-020-05685-1)
Supplement: Supplementary file 1 — Additional file 1. [file 12879_2020_5685_MOESM1_ESM.pdf]

1. Participant Details

Record ID	________________________

Interviewer Details

Interviewer Name	________________________
Date of Interview	________________________

Child's Information

Child's balanda name	________________________
Child's Yolngu First Name	________________________
Child Yolgnu surname	________________________
Child Sex	◯ Male<br>◯ Female
Do you know the child's date of birth?	◯ No<br>◯ Yes
Child Date of Birth	______________________________
Child's Age	________________________<br>(enter age as i.e. 6 mths, 1 yr)

Mother's Information

Is this interview with the mother?	◯ No<br>◯ Yes
Mother's balanda name	________________________
Mother's Yolngu First Name	________________________
Mother's Yolngu surname	________________________

Carer's Information

Carer's Yolngu Name

____

Interviewee's Residence information

Lot number of primary residence

____

2. Sociodemographic Factors

Record ID ______________________

Primary carer

Who is the main carer for the child?
- ◯ Mother
- ◯ Father
- ◯ Auntie
- ◯ Grandmother
- ◯ Sister
- ◯ Other

Mothers background information

What grade did the Mother complete at school?
- ◯ Year 7 and below
- ◯ Year 8-10
- ◯ Year 11
- ◯ Year 12
- ◯ Unknown
- ◯ Still in school

Has the mother had any training after she finished school?
- ◯ No
- ◯ Yes

What is the mothers work situation?
- ☐ Centre-link
- ☐ RJCP
- ☐ Paid employment

How well does the mother speak English?
- ◯ None
- ◯ A little bit
- ◯ Moderate amount
- ◯ Speak very well

Community Child programs

Does your child attend any of these programs?
- ☐ Baby Hub
- ☐ Families as First Teachers
- ☐ Day Centre/Child care
- ☐ Neither program

How many times per week does your child attend this program? ______________________

Smoking enviornment

Does anyone in the house smoke cigarettes?
- ◯ No
- ◯ Yes

How many people in the house smoke ______________________

3. Household factors

Record ID ______________________

Housing Information

What are the main materials of your house floor?
- ◯ Cement, tile
- ◯ Laminated material
- ◯ Fibro
- ◯ Wood
- ◯ Other

What are the main materials of the wall?
- ◯ Plaster
- ◯ Wood
- ◯ Brick
- ◯ Other

What kind of toilet does your family use?
- ◯ None
- ◯ Pit toilet
- ◯ Flush toilet

What is the main energy source your family uses for cooking?
- ☐ Firewood
- ☐ Electric
- ☐ Solar

Does your family have a working refrigerator?
- ◯ No
- ◯ Yes

Does your family have a working television?
- ◯ No
- ◯ Yes

Does your family have a working car for the household?
- ◯ No
- ◯ Yes

Does your family have a working mobile phone?
- ◯ No
- ◯ Yes

5. Hygeine assessment

Record ID	__________________________________
Do you have tap water in the house?	◯ No<br>◯ Yes
How often do you practice hand-washing (mother)?	◯ Never<br>◯ 1-2 times a day<br>◯ More than 2 times a day
Do you routinely use soap when you wash your hands? (mother)	◯ No<br>◯ Yes
What is the main reason you don't use soap?	◯ Too expensive<br>◯ None in store<br>◯ Believe unnecessary<br>◯ Other
How often does the child practice hand-washing?	◯ Never<br>◯ 1-2 times per day<br>◯ More than 2 times per day
Does the child use soap when their hands are washed?	◯ No<br>◯ Yes
Do dogs live in your house?	◯ No<br>◯ Yes
How many dogs live at the house?	__________________________________

4. Nutritional Assessment

Record ID ______________________________

Food Security

Did your household run out of food in the last two weeks and didn't have money to buy more?
- ◯ No
- ◯ Yes

In the last two weeks was there a time that your child had to miss a meal or feel hungry because there was not enough food to eat?
- ◯ No
- ◯ Yes

What was the reason this happened?
- ◯ Not enough money
- ◯ Busy, not enough time to make food
- ◯ Not enough food
- ◯ Other reason
- ◯ Don't know

Other reason ______________________________

What things do you do to get food on these days?
- ◯ Ask other families to borrow money or food
- ◯ Go hunting
- ◯ Go to Baby Hub or FAFT
- ◯ Other

Dietary habits

Does the family mostly cook at home or buy take away food?
- ◯ Cook food
- ◯ Buy take-away food
- ◯ Do both about the same

Breast-feeding patterns

Has the child ever been breast-fed?
- ◯ No
- ◯ Yes

Is the child currently breast-feeding?
- ◯ No
- ◯ Yes

What age (in months) did the child stop breast-feeding? ______________________________

Why did the child stop breast-feeding?
- ◯ Not enough milk
- ◯ Mother pregnant
- ◯ Baby doesn't want to suck
- ◯ Mother or baby too sick
- ◯ Mother too busy or at work
- ◯ Too painful
- ◯ Other

Other reason child stopped breast-feeding ______________________________

Did the child only have breast-milk for the first 6 months of life? (no other fluids like water, other drinks,or food)	◯ No<br>◯ Yes<br>◯ Can't remember
Why did the child not exclusively breast feed for 6 months?	◯ Not enough milk<br>◯ Mother pregnant<br>◯ Baby seems to need more food<br>◯ Mother or baby too sick<br>◯ Other
Other reason child wasn't exclusively breastfed?	__________________________________
Has the child ever been fed from a bottle?	◯ No<br>◯ Yes

Complementary feeding patterns

Does the child eat food?	◯ No<br>◯ Yes
What age (months) did the child start tasting food?	__________________________________

Traditional foods

Does the family eat traditional foods?	◯ No<br>◯ Yes
How often does the family eat traditional foods?	◯ Everyday<br>◯ On most days<br>◯ 2-3 days a week<br>◯ 1 day a week<br>◯ 1 day a fortnight<br>◯ Never
What type of traditional foods do you eat most often?	____
Has the child received traditional medicine before?	◯ No<br>◯ Yes
What did they receive traditional medicine for?	____
