# Supplementary material for: Enteric pathogen infection and consequences for child growth in young Aboriginal Australian children: a cross-sectional study
Source: BMC Infect Dis. 2021 Jan 6;21:9. doi: 10.1186/s12879-020-05685-1 (PMC7788727; doi:10.1186/s12879-020-05685-1)
Supplement: Supplementary file 2 — Additional file 2. [file 12879_2020_5685_MOESM2_ESM.docx]

| **ASSAY** | **TARGET** |
| --- | --- |
| Shigella | *Shigella spp.* (includes all 4 serotypes and some EIEC strains; excludes EHEC) |
| Salmonella | *Salmonella enterica* and *S. bongori* |
| Campylobacter | *Campylobacter jejuni, coli, lari,* and *doyeli;* excludes *C. hominis* |
| C.difficile | *Clostridium difficile* strains containing the toxin B gene |
| RUO: Aeromonas* | Performance Evaluation only: Aeromonas hydrophila (includes all strains) |
| Yersinia | *Yersinia spp.* (includes pathogenic strains of *Y. enterocolitica,* plus *Y. mollaretii*, *Y. intermedia*, *Y. ruckeri, Y. bercovieri* and some *Y. pestis*) |
| SaV | Sapovirus (Includes G2, G4 and some G1 (including 1.1)) |
| Shiga toxin | Shiga toxin and Shiga-like toxin 1 and 2 (from *Shigella dysenteriae* and STEC) |
| Rotavirus | Rotavirus A (includes all strains) |
| noro-1 | Norovirus Genogroup I (includes G1.1, 1.3, 1.6, 1.8, 1.9; excludes G1.2) |
| noro-2 | Norovirus Genogroup II (multiple genotypes including 2.4) |
| hAdV F,G Astrovirus | Adenovirus Group F (includes AdV40 and 41) and G (includes AdV52)  Astrovirus (includes serotypes 1 - 8) |
| Giardia (18S) | Giardia lamblia (includes assemblages A through F) |
| Cryptosporidium | *Cryptosporidium spp.* (includes *C. parvum, C. hominis, C. wrairi, C. meleagridis;* excludes *C. tyzzeri, C. baileyi, C. felis*) |
| E.histolytica | *Entamoeba histolytica* (excludes other *Entamoeba* e.g. *E. dispar* and *E. moshkovskii*) |

**Supplementary material 2.**
